# The first patient recovered from avian influenza A H7N9 viral infection: A case report and review of the literature

**DOI:** 10.1016/j.rmcr.2013.07.001

**Published:** 2013-07-31

**Authors:** Yanchao He, Qihui Huang, Jindong Shi, Zhoufang Mei, Zhijun Jie

**Affiliations:** Department of Pulmonary Medicine, The Fifth People's Hospital of Shanghai, Fudan University, China

**Keywords:** H7N9, Avian influenza A, Pneumonia, Oseltamivir

## Abstract

In March 2013, a novel avian-origin influenza A (H7N9) virus was isolated from throat swabs of 2 patients at the Fifth People's Hospital of Shanghai, China. Subsequently, 4 more patients infected by H7N9 were identified. Of the 6 patients, 4 died of acute respiratory distress syndrome. Here, we report the first case of a patient who recovered from pneumonia induced by H7N9 infection. The patient presented with fever, cough, and blood in sputum. Laboratory tests showed a low level of leukocytes, hypoxaemia, and increased levels of creatine kinase and lactate dehydrogenase. Imaging showed multiple areas of segmental ground-glass opacity in the right lung. Oseltamivir and antibiotics were administered. Supplemental oxygen helped relieve symptoms. Approximately 2 weeks after treatment, the patient finally recovered. A follow-up chest computed tomography scan taken 8 weeks later revealed that the ground-glass opacity was clearly absorbed. Therefore, timely intervention with oseltamivir and supplemental oxygen may be very important in the treatment of H7N9 infection.

## 1 Introduction

Cases of human infection with avian-origin H7 avian influenza viruses have been previously documented [1], [2], [3], [4], but infection with an N9 subtype influenza virus has not been reported in humans. Human H7 influenza infections are generally mild, causing conjunctivitis or modest respiratory symptoms. In March 2013, a novel avian-origin influenza A (H7N9) virus was identified in throat swab specimens obtained from 2 patients who died of severe pneumonia in our hospital [5]. Shortly thereafter, we discovered 4 more patients infected with H7N9. Finally, 4 of the 6 patients died, while 2 patients recovered. To the best of our knowledge, this is the first report of a patient who recovered from pneumonia induced by H7N9 infection.

## 2 Case report

A 40-year-old man who complained of ‘fever, cough, and blood in sputum persisting for 3 days’ was admitted to the Fifth People's Hospital of Shanghai, Fudan University, on 6 March 2013. He had a smoking history for 20 years (approximately 800 cigarettes/year) with an unremarkable medical history.

**Fig. 1:**
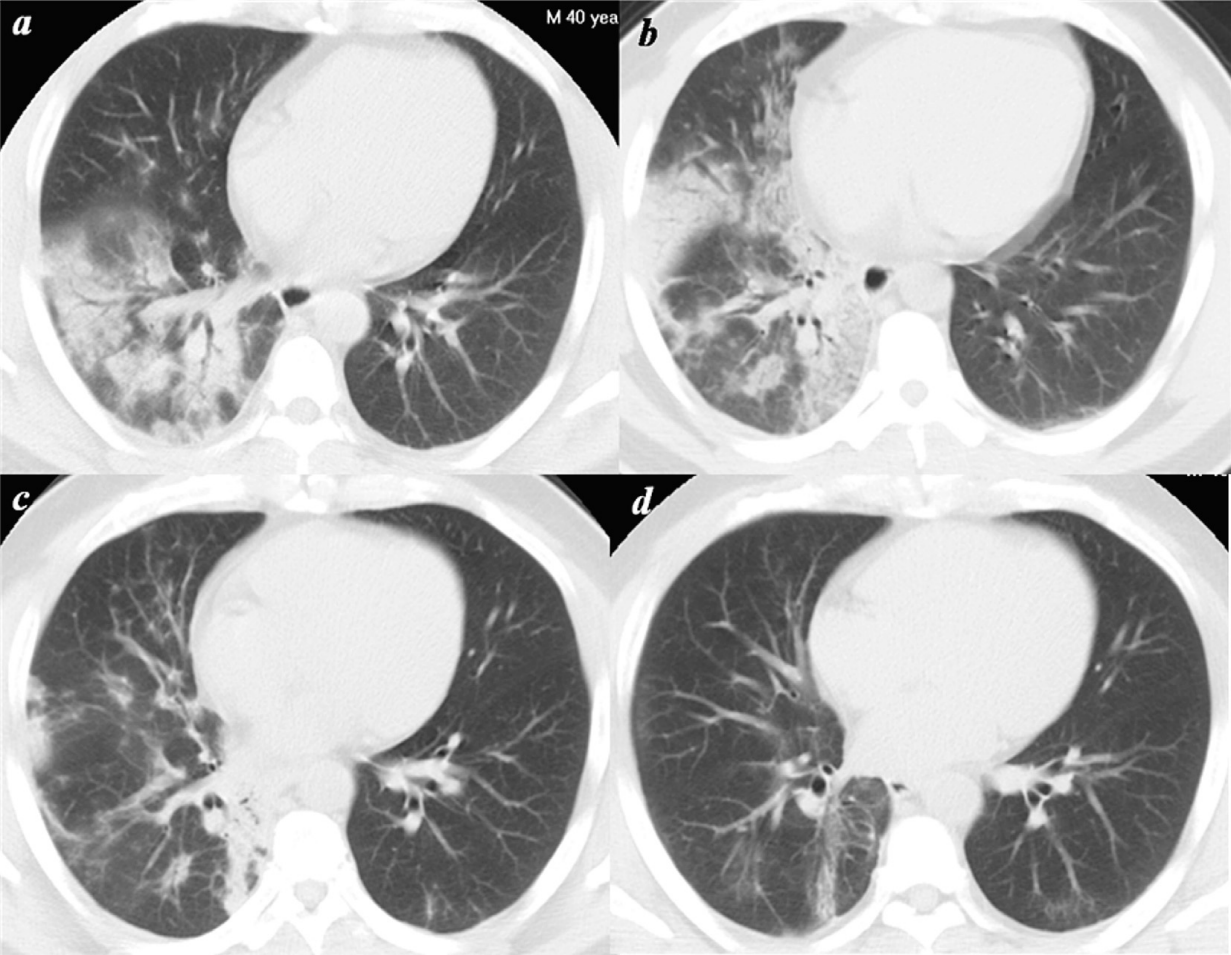
Imaging changes in the first case of recovery from H7N9. Panel a (6 March) showed multiple segmental ground-glass opacity lesions in the middle and lower lobe of the right lung with clear signs of air bronchogram. The left lung showed no pathological abnormalities, and no signs of mediastinal lymph node enlargement were seen. Panel b (10 March) showed that the opacities had become more confluent and dense, revealing a progressing inflammatory response. Panel c (17 March) showed that inflammation was absorbed compared to the findings from 10 March. Panel d (24 April) showed that inflammation was clearly further absorbed compared to previous findings.

On admission (6 March), physical examination showed a stable respiratory rate (20/min), normal blood pressure (130/80 mmHg), tachycardia (heart rate, 120 bpm), and fever (body temperature, 39 °C). Moist rales were heard in the lower lobe of the left lung. Arterial blood gas analysis revealed hypoxaemia (arterial oxygen, 64 mmHg). Blood test showed normal white blood cell (WBC) count (4.99 × 10^9^/L), while the percentage of neutrophils (78.4%) was higher than the normal range. Chest-CT showed multiple areas of segmental ground-glass opacity in the middle and lower lobe of the right lung with clear signs of air bronchogram; the left lung showed no pathological abnormality, and there were no signs of enlarged mediastinal lymph nodes (Fig. 1a). The patient was initially treated by moxifloxacin and oxygen treatment (nasal catheter oxygen inhalation with an oxygen flow rate of 4 L/min).

On the day after admission (7 March), laboratory tests showed elevated enzyme levels: creatine kinase (CK), 984 U/L, creatine kinase isoenzymes (CK-MB), 20 U/L, lactate dehydrogenase (LDH), 498 U/L, alanine aminotransferase (ALT), 46 U/L, and aspartate aminotransferase (AST), 51 U/L. The patient's body temperature did not return to normal. We continued to obverse him while providing physical cooling.

On day 2 after admission (8 March), the patient's clinical symptoms were not resolved, and his body temperature increased to 39.7 °C. Hypoxaemia persisted after inhaling oxygen (arterial blood gas analysis: pH 7.5; arterial partial pressure of carbon dioxide, 30 mmHg; arterial partial pressure of oxygen, 64 mmHg) (Table 1). We replaced moxifloxacin with meropenem as antibacterial therapy.

**Table 1:** Laboratory data of the first case of recovery from H7N9.

Date	6 Mar	7 Mar	8 Mar	10 Mar	11 Mar	13 Mar	18 Mar	30 Mar	23 Apr	Normal range
WBC (×10^9^/L)	NA	4.49	4.24	3.38↓	NA	7.77	6.23	9.86	7.18	4.0–10.0
Lym (×10^9^/L)	NA	1.08	0.94	1.17	NA	1.51	1.71	3.92	2.92	1.0–4.0
PLT (×10^9^/L)	NA	104	113	144	NA	232	370	181	201	100–400
LDH (U/L)	NA	498↑	NA	NA	NA	NA	263	NA	NA	135–215
CK (U/L)	NA	984↑	NA	NA	NA	NA	NA	NA	NA	24–192
CK-MB (U/L)	NA	20	47	35	NA	33	NA	NA	NA	0–25
ALT (U/L)	NA	46	54↑	217↑	NA	155↑	100↑	81↑	39	0–55
AST (U/L)	NA	51↑	71↑	160↑	NA	54↑	34	34	25	0–50
pH	7.51	NA	7.5^†^	NA	7.42	NA	NA	NA	NA	7.35–7.45
PaCO_2_ (mmHg)	28	NA	30^†^	NA	35	NA	NA	NA	NA	35–45
PaO_2_ (mmHg)	64↓	NA	64↓^†^	NA	113	NA	NA	NA	NA	83–108

WBC: white blood cells, Lym: lymphocyte, PLT: platelets, LDH: lactate dehydrogenase, CK: creatine kinase, CK-MB: creatine kinase isoenzyme, ALT: alanine aminotransferase, AST: aspartate aminotransferase, PaO2: arterial partial pressure of oxygen, PaCO2: arterial partial pressure of carbon dioxide.

† After nasal catheter oxygen inhalation with oxygen flow 4 L/min.

↓ Lower than reference value.

↑ Higher than reference value.

After 4 days of treatment (10 March), the patient's clinical symptoms were not resolved. A blood test revealed a decreased WBC count of 3.38 × 10^9^/L, and increased levels of ALT of 217 U/L and AST of 160 U/L (Table 1). Chest-computed tomography (CT) showed that the opacities had become more confluent and dense, with a new large field of opacities in the right lung and patchy opacities in the lower lobe of the left lung. Inflammation had also clearly progressed (Fig. 1b). On the same day, a 27-year-old male patient died of progressive pneumonia and acute respiratory distress syndrome. After we discussed the similar features of the 2 cases and took flu season into account, oseltamivir and amantadine were administered as antiviral therapy.

On day 11 (17 March), the patient's clinical symptoms had resolved except for occasional cough and blood in sputum. The patient's body temperature had decreased to within the normal range. SpO_2_ rose to 98%. Chest-CT scanning showed that inflammation was absorbed compared to the findings from 10 March, but irregular opacities remained in the mid-lower lobe of the right lung (Fig. 1c). On 18 March, the patient was discharged from the hospital.

On 24 April, the patient came back for follow-up. CT scanning clearly showed that inflammation was further absorbed compared to earlier findings (Fig. 1d). Blood cell counts and liver function tests were within the normal ranges (Table 1).

We tested the patient's pulmonary function during his hospitalization (11 March) and found restricted pulmonary ventilation disorder (FEV1%:45%, FEV1/FEVC%:102%, FVC%:48) and diffuse dysfunction (DLCO%:49%). Approximately 5 weeks after the patient was discharged from the hospital (24 April), pulmonary function tests became normal (FEV1%: 94%, FEV1/FVC%:114%, FVC%:86%. DLCO%:85).

## 3 Discussion

To the best of our knowledge, this is the first report of a patient who recovered from pneumonia caused by a lethal case of human avian-origin influenza virus H7N9. H7N9 virus was not found in the throat swab specimens obtained from this patient on 8 March; however, specific viral antibody IgG was detected in recovery serum (IgG:1:40) at the Chinese Centre for Disease Control and Prevention on 13 April. Therefore, this patient was confirmed to be infected with H7N9. The patient complained of fever, cough, and blood in sputum and presented with decreased WBC count after virus infection. Blood test showed increased enzyme levels (LDH, CK, CK-MB, ALT, and AST), with especially high levels of LDH and CK. Chest-CT revealed ground glass changes, and hypoxaemia was noticed after admission, suggesting high H7N9 viral virulence.

Antibacterial therapy did not yield positive results in the rapid progression of the disease. We considered the possibility of influenza virus infection. Oseltamivir and amantadine were administered as antiviral therapy on day 4 after admission. Although we did not use oseltamivir and amantadine in the first 48 h, clinical symptoms had significantly remitted. However, a 27-year-old male patient who was also positive for H7N9 died after active treatment for 6 days. Therefore, the prognosis of human H7N9 infection may be related to the viral load of H7N9, autoimmunity, and intervention time. The 27-year old patient was a pork trader in the live-poultry market and was admitted to the hospital almost a week after illness onset, during which time he was also actively positive for hepatitis B.

The pathogenesis of human avian-origin influenza A (H7N9) virus infection is unknown. It has been previously proposed that highly pathogenic avian influenza virus infections lead to the induction of a strong inflammatory response, characterized by elevated serum levels of cytokines and chemokineses [6]. This view may be a breakthrough for us to explore the pathogenesis of avian-origin influenza virus in human. In H5N1 and H9N2 influenza viruses, neuraminidase stalk length was associated with virulence and pathogenesis in mice [7], [8]. In addition, a mutation at position 627 of the gene encoding the PB2 protein, which is associated with the outcome of infection in mice [9], was found to be associated with the virus [1]. We speculated that this mutation may contribute to the rapid progression of the disease in patients. An epidemiological survey on H7N9 in China revealed that among 82 patients, 76% had a history of exposure to poultry [10]. Although the patient is this report denied a history of exposure to poultry, H7N9 virus was found in the poultry of 2 neighbouring markets. No symptoms were observed in the hospital among doctors and nurses caring for the patient, suggesting that the disease does not readily spread. Moreover, limited human-to-human transmission was observed in the H7 outbreak in the Netherlands in 2003 [3]. However, we do not exclude the possibility of human-to-human transmission.

## 4 Conclusion

In conclusion, avian influenza H7N9 infection remains a new disease entity. Factors such as clinical symptoms with fever and cough; laboratory tests with low levels of leukocytes, hypoxaemia, and increased enzyme levels; and chest-CT showing multiple areas of segmental ground-glass opacity as well as a history of direct contact with poultry are criteria for the diagnosis of avian influenza H7N9 infection. Furthermore, timely intervention with oseltamivir and supplemental oxygen may be very important therapies for H7N9 infection. Future studies are needed to further characterize the disease, as well as elucidate the molecular and biological characteristics of patients infected with H7N9 and their prognostic significance, so as to devise optimal treatment strategies.

## Funding information

This work was supported by grants from the Shanghai Committee of Science and Technology (No. 134119b1200), Shanghai Health Bureau Scientific Research (20114307), and the training program for young doctors foundation of Shanghai Municipal Health Bureau.

## Financial/nonfinancial disclosures

The authors disclose to Respiratory Medicine that no potential conflicts of interest exist with any companies/organizations whose products or services may be discussed in this article.

## Authors' contributions

Dr. Jie outlined the case report and organized the writing. Other physicians of the Fifth People's Hospital of Shanghai treated the patient and provided the first-hand material for the case report. Dr. He drafted this manuscript.

## Conflict of interest statement

The authors express their gratitude to the Fifth People’s Hospital of Shanghai, Fudan University for providing medical resources in the case report. The authors have reported to Respiratory Medicine that no potential conflicts of interest exist with any companies/organizations whose products or services may be discussed in this article.
